# Preliminary monitoring of knockdown resistance (*kdr*) mutation in *Anopheles stephensi*: insights from a malarious area in Southeastern Iran

**DOI:** 10.1186/s12936-024-05042-6

**Published:** 2024-07-17

**Authors:** Alireza Sanei-Dehkordi, Azim Paksa, Mohammad Amin Gorouhi, Moussa Soleimani-Ahmadi, Seyed Aghil Jaberhashemi, Yaser Salim Abadi

**Affiliations:** 1 Infectious and Tropical Diseases Research Center, Hormozgan Health Institute, Hormozgan University of Medical Sciences, Bandar Abbas, Iran; 2 Department of Biology and Control of Disease Vectors, Faculty of Health, Hormozgan University of Medical Sciences, Bandar Abbas, Iran; 3 Department of Biology and Control of Disease Vectors, School of Health, Shiraz University of Medical Sciences, Shiraz, Iran; 4 Department of Vector Biology and Control, Faculty of Public Health, Kerman University of Medical Sciences, Kerman, Iran; 5 Department of Health Services and Health Promotion, School of Health, Rafsanjan University of Medical Sciences, Rafsanjan, Iran

**Keywords:** *Anopheles stephensi*, Malaria, Knockdown resistance(*kdr*) mutation, Iran

## Abstract

**Background:**

*Anopheles stephensi* is recognized as the main malaria vector in Iran. In recent years, resistance to several insecticide classes, including organochlorine, pyrethroids, and carbamate compounds, has been reported for this medically important malaria vector. The main objective of the present study was to evaluate the insecticide susceptibility status of *An. stephensi* collected from the southern part of Iran, and to clarify the mechanism of resistance, using bioassay tests and molecular methods comparing the sequence of susceptible and resistant mosquitoes.

**Methods:**

Mosquito larvae were collected from various larval habitats across six different districts (Gabrik, Sardasht, Tidar, Dehbarez, Kishi and Bandar Abbas) in Hormozgan Provine, located in the southern part of Iran. From each district standing water areas with the highest densities of *Anopheles* larvae were selected for sampling, and adult mosquitoes were reared from them. Finally, the collected mosquito species were identified using valid keys. Insecticide susceptibility of *An. stephensi* was tested using permethrin 0.75%, lambdacyhalothrin 0.05%, deltamethrin 0.05%, and DDT 4%, following the World Health Organization (WHO) test procedures for insecticide resistance monitoring. Additionally, knockdown resistance (*kdr*) mutation in the voltage-gated sodium channel (*vgsc*) gene was sequenced and analysed among resistant populations to detect possible molecular mechanisms of observed resistance phenotypes.

**Results:**

The susceptibility status of *An. stephensi* revealed that resistance to DDT and permethrin was found in all districts. Furthermore, resistance to all tested insecticides in *An. stephensi* was detected in Gabrik, Sardasht, Tidar, and Dehbarez. Analysis of knockdown resistance (*kdr*) mutations at the *vgsc* did not show evidence for the presence of this mutation in *An. stephensi*.

**Conclusion:**

Based on the results of the current study, it appears that in *An. stephensi* from Hormozgan Province (Iran), other resistance mechanisms such as biochemical resistance due to detoxification enzymes may be involved due to the absence of the *kdr* mutation or non-target site resistance. Further investigation is warranted in the future to identify the exact resistance mechanisms in this main malaria vector across the country.

## Background

Iran is a malaria-endemic country. Seven species of *Anopheles* mosquitoes are recognized as malaria vectors, namely *Anopheles culicifacies *sensu lato (*s.l.*), *Anopheles fluviatilis s.l.*, *Anopheles stephensi*, *Anopheles superpictus s.l.*, *Anopheles maculipennis s.l. Anopheles dthali*, and *Anopheles sacharovi.* Among these, *An. stephensi* is notably recognized as the main malaria vector [1–4]. The majority of malaria cases are concentrated in the southern and southeastern provinces of Iran including Hormozgan, Sistan, and Baluchestan, and southern parts of Kerman. These regions are characterized by refractory malaria, and have suitable conditions for the reproduction of *Anopheles* mosquitoes; most of the aforementioned malaria vectors are present there [4–6]. Various insecticides, such as malathion, mirimiphos-methyl, DDT, dieldrin, propoxur, lamdacyhalothrin and deltamethrin, belonging to different groups, have been utilized for controlling *Anopheles* mosquitoes in malaria-endemic areas. This control is implemented through different intervention tools, including indoor residual spraying (IRS) and insecticide-treated nets (ITNs). However, the extensive use of insecticides has led to the emergence of resistance in *An. stephensi* against most of these insecticides [7, 8]. Reports from different districts of Hormozgan province, such as Siahoo, Geno, Bandar Abbas, Bashagard, and Jask, have indicated resistance in *An. stephensi* to DDT and lambdacyhalothrin through the World Health Organization (WHO) insecticide susceptibility protocol [9–12]. This resistance is attributed to mutations in the voltage-gated sodium channel (vgsc), resulting in insecticide target site insensitivity, known as knockdown resistance (*kdr*) mutations. Additionally, metabolic resistance due to detoxification enzymes, such as cytochrome P450 monooxygenases (P450s), is implicated in pyrethroid metabolic resistance in *An. stephensi* [13–15]. For instance, biochemical and molecular investigations conducted on *An. stephensi* from Afghanistan revealed the presence of both *kdr* mutation and metabolic mechanisms responsible for insecticide resistance [16]. Although there are several reports about emergence of resistance to insecticide in *An. stephensi* from different parts of Hormozgan province, but no study has been conducted on this species to detect the mechanisms of resistance to insecticides therefore the objective of the current study was to characterize the susceptibility status of the main malaria vector, *An. stephensi*, as well as its resistance mechanisms, utilizing bioassay tests and molecular methods in the southern part of Iran.

## Methods

### Study area

**Fig. 1:**
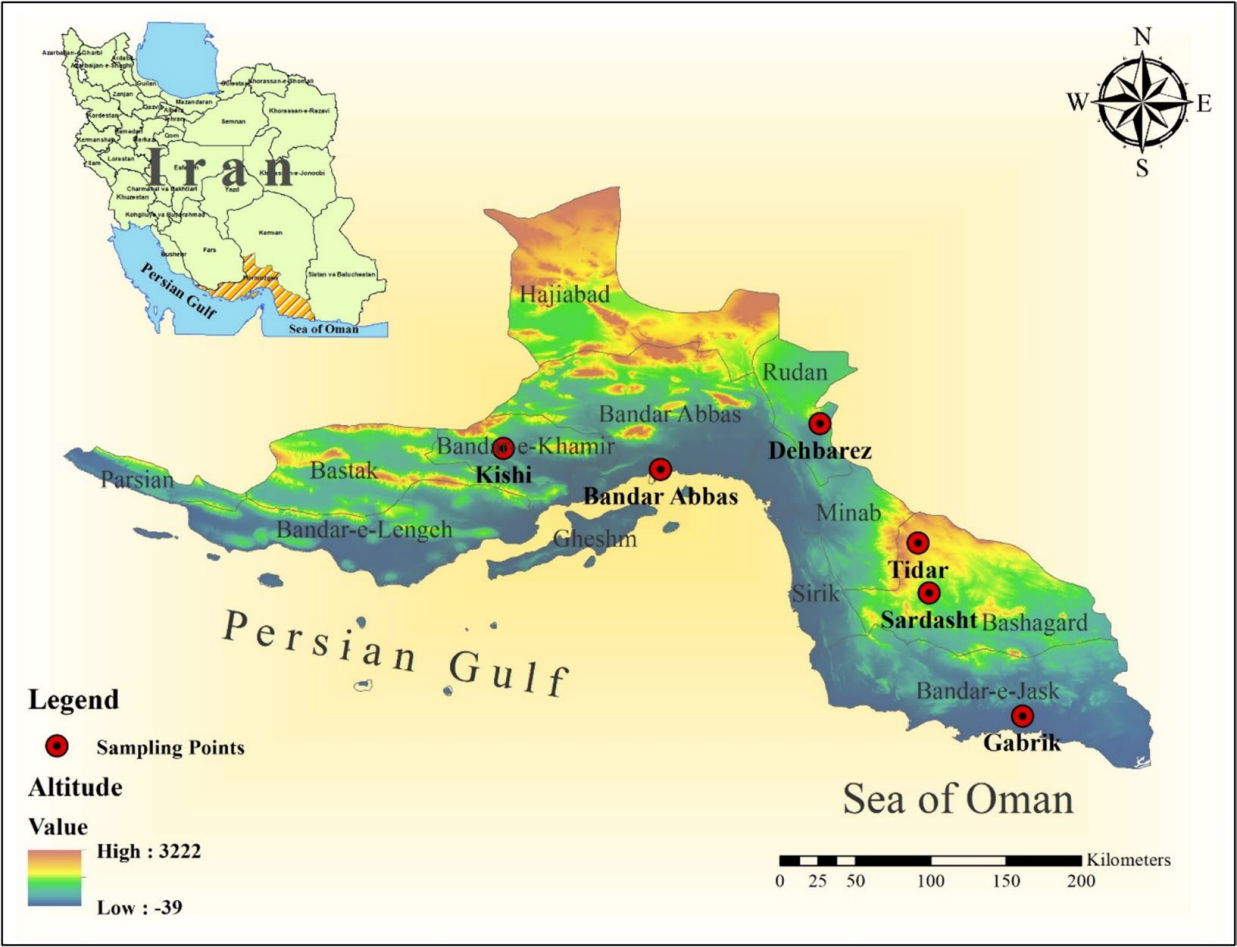
Geographical locations of collection sites of *Anopheles stephensi*. Map was constructed using Arc-GIS software, version 10.8 (ESRI, Redlands, CA, USA)

Hormozgan Province is localized in the southern region of Iran, bordering the Persian Gulf. It encompasses 13 major cities and lies between latitude 25°24′–28°53′N and longitude 52°44′–59°14′ E [17]. For the present study, six cities (Gabrik, Sardasht, Tidar, Dehbarez,Kishi and Bandar Abbas) within Hormozgan Province, which have a history of implementing malaria control programs using IRS and ITNs, were selected for entomological studies (Fig. 1).

### Sample collection

**Fig. 2:**
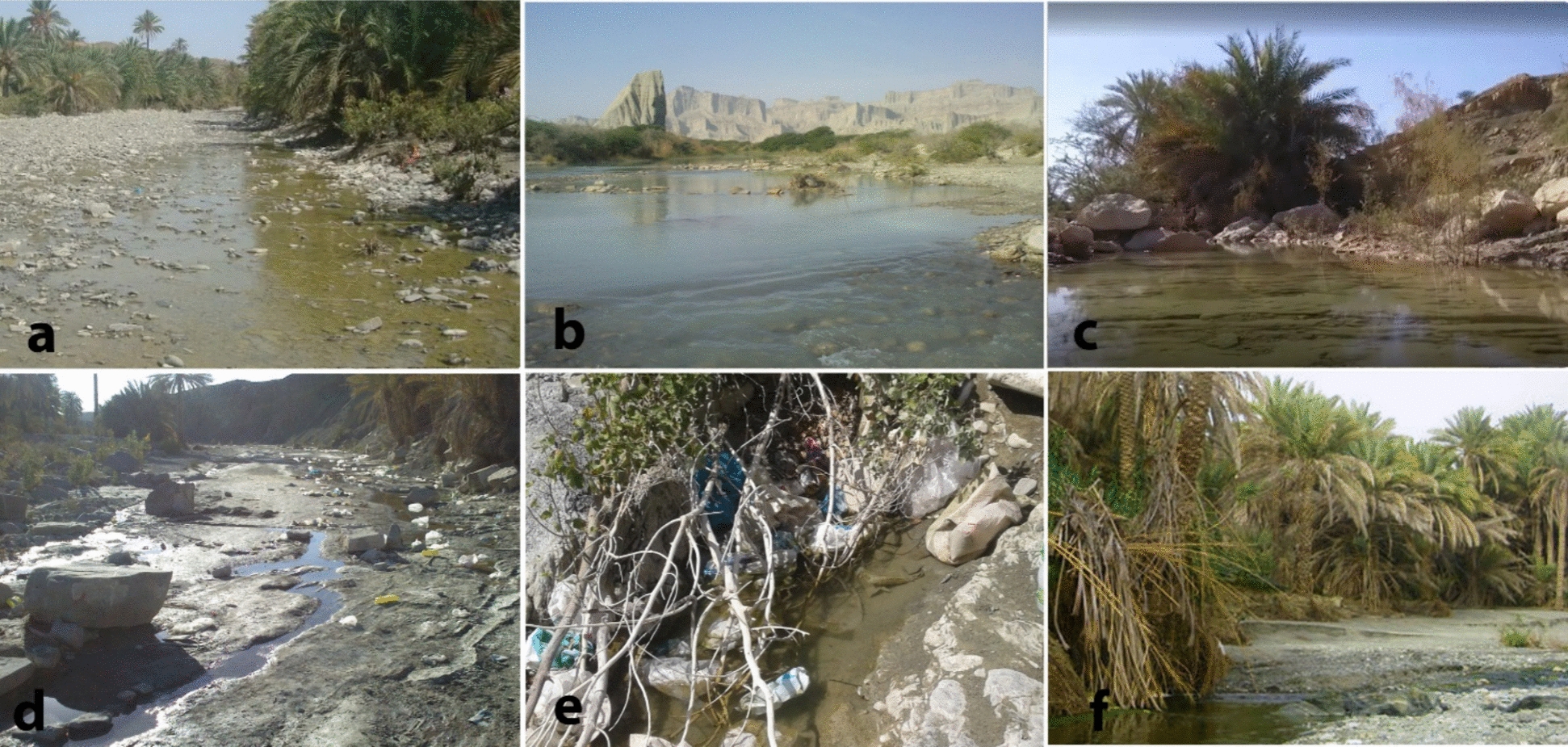
Larval habitats of *Anopheles stephensi* in Hormozgan Province, Iran. a: Tidar, b: Gabrik, c: Kishi, d: Sardasht, e: Bandar Abbas, f: Dehbarez

Six distinct sampling locations were chosen across five counties: Jask, Bashagard, Bandar Abbas, Khamir, and Rudan. These areas have a historical record of insecticide use in malaria control programs. The topographical and climatic conditions of these districts present a diverse environmental landscape. Jask, with its hot desert climate, is indicative of its arid summers and mild winters. Bashagard, characterized by its mountainous terrain, endures cold winters and hot, dry summers, with a climate moderated by its proximity to the mountains. Bandar Abbas and Khamir, situated at a lower elevation, are subject to a subtropical desert climate, reflecting it’s hotter and more humid conditions. Rudan's climate mirrors that of Bandar Abbas but with longer, sweltering summers and short, cool winters, mostly clear skies (Fig. 2).

Mosquito larvae were collected from various larval habitats using the standard dipper method [18]. From each district standing water areas with the highest densities of *Anopheles* larvae were selected for sampling. Collected samples were transferred to the insectary and placed in a holding container for rearing under standard conditions with a temperature range of 25–29 °C, a photoperiod of 12:12 h (light: dark), and a humidity level of 50–70%. The emerged adult mosquitoes were identified using valid keys [19] and were fed with 10% aqueous sucrose solution and subsequently utilized in both bioassay and molecular investigations.

### Adult susceptibility tests procedure

Adult susceptibility tests of mosquitoes were conducted following WHO guidelines, utilizing standard insecticide-impregnated filter paper: permethrin 0.75%, lambdacyhalothrin 0.05%, deltamethrin 0.05%, and DDT 4%. Each bioassay involved 100 test mosquitoes (four replicates of 25), non-blood-fed female mosquitoes aged between 3 and 5 days, Additionally, 50 female control mosquitoes (2 replicates) were exposed to the insecticide-impregnated filter paper for 1 h. After a 24-h recovery period, the mortality rates were recorded, moreover, mosquitoes were supplied with 10% fresh sugar solution during this time. Correction of the mortality rate in the test samples was corrected using the Abbott formula when the mortality rate of control ranged between 5 and 20%. Tests with a control mortality rate exceeding 20% were repeated [20]. Based on WHO criteria, the susceptibility level of the mosquitoes was categorized into three classes: mortality between 98–100% indicated susceptibility; mortality between 90 and 97% suggested a candidate for resistance or tolerance requiring further investigation to confirm resistance; and mortality less than 90% was classified as resistance [21].

### Molecular analysis of the voltage-gated sodium channel (*vgsc*) gene

The genomic DNA from mosquito legs was extracted using the Collins extraction method [22]. Polymerase chain reaction (PCR) assays were developed for amplifying the S6 segment of domain II of the *vgsc* gene. This fragment contains a region in the exon that leads to *kdr* type resistance in case of mutation. The forward and reverse primers used were St-F (5′- GAT TGT GTT CCG TGT GCT GT -3′) and St-L/SR (5′- GCG GGC AGGGCG GCG GGG GCG GGG CCC GAT CGG AAA GTA AGT TAC TTA CGT CT -3′), respectively.

The cycling conditions consisted of an initial denaturation step at 95 °C for 5 min, followed by 35 cycles at 95 °C for 30 s, 48 °C for 30 s, and 72 °C for 45 s, and a final extension step at 72 °C for 7 min [23]. From each resistant population extracted DNA with high quality send for sequencing after that sequences obtained were submitted as queries to the National Center for Biotechnology Information’s (NCBI) Basic Local Alignment Search Tool (BLAST) to confirm correct loci were amplified. A comparison of the sequence in susceptible and resistant mosquitoes was conducted on all samples to detect the presence or absence of *kdr* type resistance in *vgsc*, referencing sequence details from a previous study by Singh et al. [23] on *An. stephensi*. Finally, all sequences were deposited in the gene bank with accession numbers (MN868413.1- MN868424.1).

## Results

### Insecticide susceptibility status

**Fig. 3:**
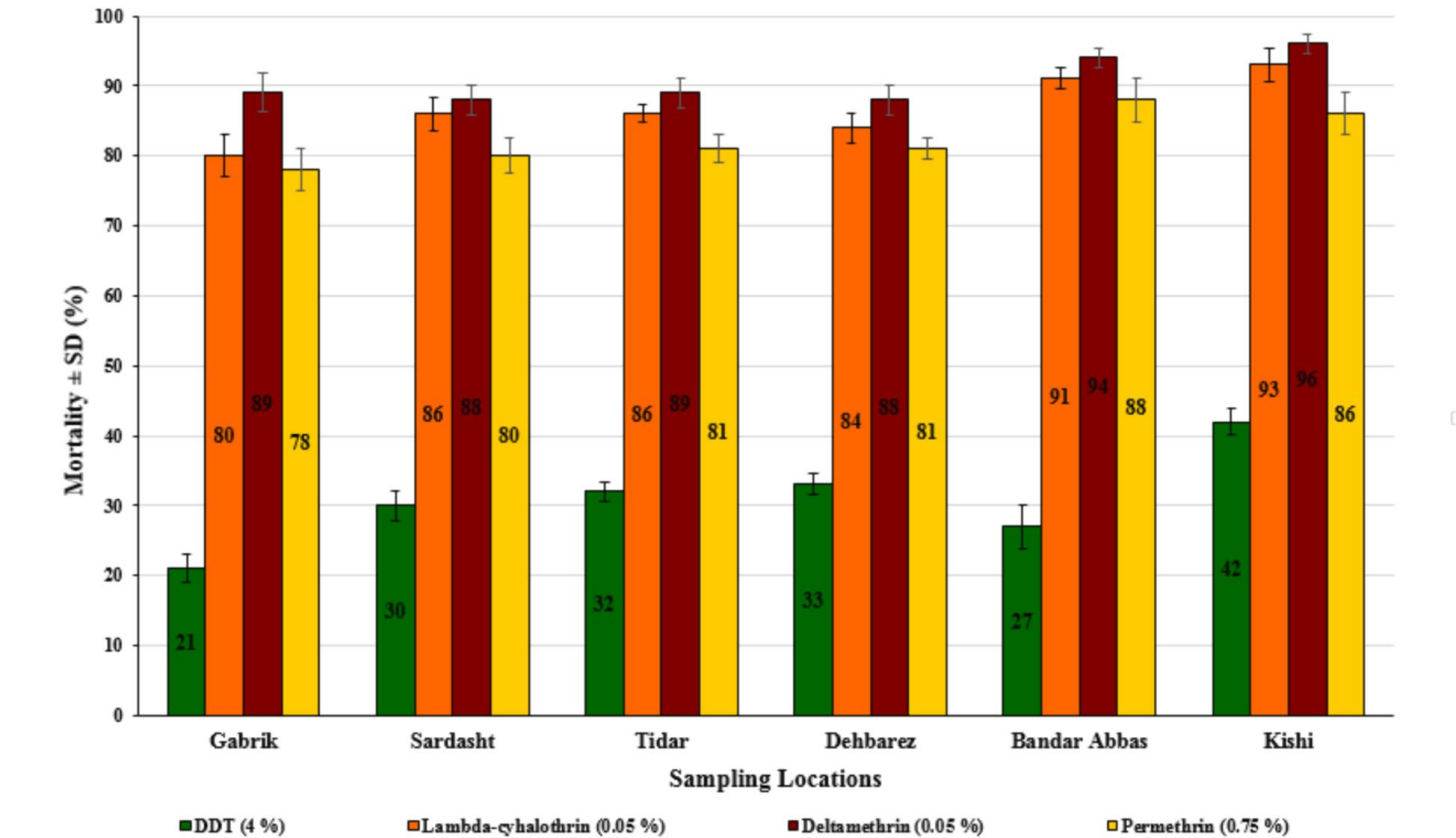
The mortality rates in *An. stephensi* exposed to different insecticides

The mortality rate of *An. stephensi* following exposure to the insecticides was calculated after a 24-h recovery period and is illustrated in Fig. 3. According to the criteria for insecticide resistance outlined by the WHO, resistance to DDT and permethrin have been observed across all districts. However, it is noteworthy that while resistance to all tested insecticides in *An. stephensi* was detected in Gabrik, Sardasht, Tidar, Dehbarez, a different pattern emerged in Kishi and Bandar Abbas. In Kishi and Bandar Abbas, mosquito mortality was observed between 90–97% for lambdacyhalothrin and deltamethrin, indicating the population of these areas as resistance candidates. Across all districts, DDT exhibited the lowest toxicity in *An. stephensi,* suggests a high level of resistance to this insecticide.

### Analysis of *vgsc* sequence

**Fig. 4:**
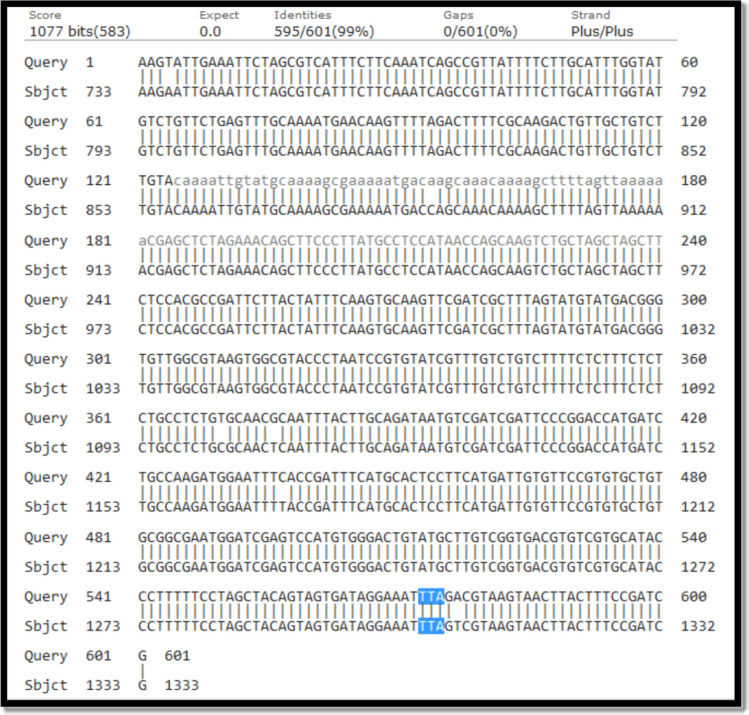
Comparing the sequence of *An. stephensi* (resistant strain) with the reference strain from Gene Bank as susceptible

Based on adult susceptibility test results in Gabrik, Sardasht, Tidar, and Dehbarez, resistance to all tested insecticides was detected. Subsequently, molecular investigations were carried out on their population. The sequences of *vgsc* in all populations exhibited a high degree of similarity (99%) with the sequences from India (accession number: JF304954.1), moreover, these sequences correspond to a susceptible strain that was deposited in Gene Bank [23]. The comparison of the region containing *kdr* mutations at the locus L1014 (blue highlighted in Fig. 4) due to the amino acid substitution of leucine (TTA) with phenylalanine (TTT) or serine (TCA) in the *vgsc* gene. *kdr* mutations L1014F and L1014S [23, 24] were not found in resistant populations across all districts.

## Discussion

The findings of the current study indicated resistance to DDT detected in *An. stephensi* across all studied areas. This corroborates with previous studies conducted in the southern part of Iran, where a high level of resistance to DDT in *An. stephensi* has been observed [25–27], including in Hormozgan Province [7, 9–12]. Moreover, resistance to DDT in *An. stephensi* has been reported in neighboring countries of Iran, such as Pakistan, Saudi Arabia, Iraq, Oman, United Arab Emirates, and Afghanistan [12, 13, 15]. Although resistance to all pyrethroid insecticides, as well as DDT, was detected in *An. stephensi* in Gabrik, Sardasht, Tidar and Dehbarez, this species exhibited tolerance to lambdacyhalothrin and deltamethrin in Bandar Abbas and Kishi. In a previous study conducted by Zare et al*.* in Jask County, recognized as an active malaria focus in Hormozgan Province, insecticide susceptibility tests revealed that *An. stephensi* was resistant to lambda-cyhalothrin and tolerance to Deltamethrin. Additionally, they reported that this observation might be attributed to the utilization of these mentioned insecticides for IRS and LLINs. Although concerning permethrin, the results differed, as *An. stephensi* was found to be susceptible to it [12].

The susceptibility status of *An. stephensi* to tested insecticides underwent alterations, particularly with an increase in resistance to deltamethrin, a change also noted for permethrin, which had been previously considered susceptible. In the current investigation, insecticide susceptibility tests revealed that *An. stephensi* demonstrated resistance to Permethrin in Gabrik district as one of the regions in Jask county. Furthermore, this emergence of insecticide resistance was also attributed to the aforementioned intervention measures for controlling malaria vectors. Reports of resistance to pyrethroid compounds have also been documented in Southeast Iran concerning *An. stephensi* against cyfluthrin and lambdacyhalothrin, mirroring this study. The usage of IRS and ITNs has been implicated in the escalation of this resistance status [1]. Resistance to pyrethroids insecticides in a malarious area where IRS and ITNs are used should be considered an alarm signal, as the development of resistance to insecticides leads to a reduction of the impact of vector control actions [28, 29]. Analysis of the *vgsc* gene indicated the absence of *kdr* L1014F and L1014S mutations in all studied areas. This suggests the presence of other resistance mechanisms, such as metabolic resistance due to detoxification enzymes, in *An. stephensi.* Similar findings have been reported in the Somali region of eastern Ethiopia, where researchers observed the absence of *kdr* L1014F and L1014S mutations in the collected samples of *An. stephensi*. These researchers have suggested that the resistance to pyrethroids observed in the species may be due to metabolic or other mechanisms [14].

In two previous studies in Sistan and Baluchestan, the neighbouring province of Hormozgan, different types of resistance mechanisms were identified in *An. stephensi* populations. Initially, biochemical analyses revealed metabolic mechanisms involved in cyfluthrin and DDT resistance in *An. stephensi* from Chabahhar region [6]. Subsequently, molecular assays were conducted in Saravan region, on *An. stephensi* populations with tolerance to deltamethrin, permethrin and resistance to DDT, which provided evidence for *kdr* mutation among examined samples. Furthermore, following the initial detection of *kdr* allele from a pyrethroid-selected strain in Dubai, the presence of the L1014F mutation, the same mutation previously described, was also reported from the Saravan region, in Iran [28, 30]. In eastern Afghanistan, regarding *An. stephensi*[16] and in West Africa concerning *An. gambiae* [31], the findings indicated that both of these mechanisms are involved in resistance. Similar to these findings, it is plausible that the same mechanisms may also exist in the aforementioned populations from Sistan and Baluchestan. However, because each of these studies examined only one resistance mechanism separately, their results differed.

It is noteworthy that most studies in Iran have primarily focused on biochemical analyses to elucidate resistance mechanisms in malaria vectors, with only a few examining both biochemical and molecular mechanisms together. Additionally, the overactivity of detoxification enzymes has been frequently implicated in resistance in malaria vectors [6, 32]. For instance, in a temephos-resistant strain of *An. stephensi* from Chabahar, molecular analysis did not reveal evidence of G119S mutation in the acetylcholinesterase gene, but biochemical assays indicated enzymatic involvement in resistance [33].

## Conclusion

In the present study, resistance against all tested insecticides was observed in *An. stephensi* in most of the studied areas. The absence of the *kdr* mutation in resistant populations suggests that the observed resistance may be attributed to biochemical or metabolic mechanisms. This main malaria vector in Iran has demonstrated resistance to all tested insecticides, indicating the need for further biochemical studies to precisely identify the resistance mechanisms in *An. stephensi*.

## Data Availability

The data supporting the findings of the study must be available within the article and/or its supplementary materials, or deposited in a publicly available database.
